# A New Subscriber

**Published:** 1901-07

**Authors:** 


### A NEW SUBSCRIBER.

He said his name was Bugg; B-u-g-g. *Doctor* Bugg from Buggville. He said he "seen the *Red Back* coated in The Brief," and "bein' in town he thought he'd subscribe,"—"tho,' " he added, "I git more litera-*chure* than I kin read; sample copies."

I told him that was right. He ought by all means to keep up with the procession, and as the *Red Back* was the band and the band wagon, if he'd get up on the seat with me he'd hear the music all the way there and back.

He glared at me.

I wrote him out a receipt and handed it to him, rubbing my hands in anticipation of the dollar. I hadn't seen one in a week.

He said he'd send the money this fall.

I asked him to take a seat. He took a seat; he took the cushioned arm chair.

He said it was warm.

I told him to forget it; to not let a little thing like that worry him; and we shouldn't let trifles make us unhappy in this world.

He glared at me again.

A big mouthed shallow fellow had been in on me just ahead of Bugg, and had nearly talked me to death. That is, he didn't let *me* talk any, and that always makes me tired. I was thinking of the 'possum story as applicable to my recent caller, when Bugg come in, and as he didn't seem to be in a talking humor, and I was, I lit in and told him the 'possum story. I told him a fellow named Jones put a little bell with a handle to it and a knob on top in his hen house to scare the 'possum that was getting a frying size pullet every night. The bell scared the 'possum all right, but he didn't stay scared; he soon discovered that there was no danger, so, after reconnoitering he took down the bell and carefully examined it. "Well," he said, "I don't know what you *are,* but you have got the biggest *mouth,* and the longest *tongue,* and the smallest *head*—"

Here Bugg turned a cold, fishy eye on me, and said:

"That ain't the way I heard it. The feller's name wan't Jones, it were Johnson; and 'twant nary 'possum; hit were a *mink.*"

And he told the story over, *verbatim,* with those two changes.

I like for fellers to correct me when I don't tell jokes just right. I'll learn by and bye.

Bugg picked up the book I had been reading. It was "Bacon *versus* Shakespeare,"—(the controversy as to who wrote Shakespeare).

"Like Bacon and Shakespeare?" I said. "Ever read it?"

"Naw," said Bugg, with a fishy stare, "I don't care much for *Shakes*peare, but I am very fond of Bacon."

And he swallowed. I saw his goozle go up and down. He thought I meant breakfast bacon, for, with a far away look he thoughtfully added, "sliced real thin and br'iled."

Now, what pesters me, is to find out which of us was the fool, and if that fellow was not guying me, after all.

